# Postoperative delirium is a risk factor for complications and poor outcome after total hip and knee arthroplasty

**DOI:** 10.1080/17453674.2021.1980676

**Published:** 2021-10-05

**Authors:** Matthias Meyer, Julia Götz, Lukas Parik, Tobias Renkawitz, Joachim Grifka, Günther Maderbacher, Tobias Kappenschneider, Markus Weber

**Affiliations:** a Department of Orthopaedic Surgery, Regensburg University Hospital, Bad Abbach, Germany;;; b Heidelberg University Orthopedic Hospital, Heidelberg, Germany

## Abstract

Background and purpose — Improving health care and demographic change have resulted in a steady increase in geriatric patients undergoing total hip (THA) and knee (TKA) arthroplasty. Postoperative delirium (POD) is a frequent and severe complication after major surgery. Therefore, we analyzed the impact of POD on outcome after THA and TKA.

Patients and methods — In a consecutive series of 10,140 patients who had undergone elective THA or TKA between 2011 and 2020, rates of reoperation within 90 days, readmission within 90 days, complications, and responder rate as defined by the OMERACT-OARSI criteria were compared between patients with and without POD. Multivariable logistic regression models were used to assess the relationship between POD and other postoperative complications.

Results — Patients with POD showed higher rates of reoperation (12% vs. 5%), readmission (15% vs. 5%), surgical complications (7% vs. 2%), non-surgical complications (8% vs. 4%), Clavien–Dindo IV° complications (10% vs. 2%) and transfusion (14% vs. 2%). POD led to lower responder rate (76% vs. 87%) 1 year after total joint replacement. All previous comparisons statistically significant. Multivariable logistic regression analyses revealed POD as an independent risk factor for reoperation (OR = 2; CI 1–3), readmission (OR = 2; CI 2–4) and Clavien–Dindo IV° complications (OR = 3; CI 2–5).

Interpretation — POD is a serious problem in elective joint replacement. Affected patients suffer more complications and show poor patient-reported outcome 1 year postoperatively. Systematic prevention strategies and standardized therapy protocols are mandatory to avoid burden to patients and healthcare providers.

Numbers of primary total hip and knee arthroplasty (THA and TKA) are projected to grow 71% and 85%, respectively, by 2030 (Sloan et al. 2018). Demographic change as well as improvement in general living standards, health care, nutrition, and education result in a steady increase in geriatric patients undergoing major surgery, such as THA and TKA (United Nations et al. 2017).

With an incidence of 0.7% to 2.2%, postoperative delirium (POD) is a common complication in patients undergoing elective THA and TKA (Petersen et al. 2017, Aziz et al. 2018, Weinstein et al. 2018, Yang et al. 2020). POD is considered to be a potent risk factor for adverse events, such as prolonged length of stay and discharge to nursing facility (Petersen et al. 2017, Aziz et al. 2018). Former studies showed that patients with POD had up to 3-fold increased length of stay and complication rates (Petersen et al. 2017, Aziz et al. 2018). Although delirium has been described in the medical literature for more than 2 millennials, especially hypoactive forms of POD are still frequently not recognized and managed appropriately in daily practice (Inouye et al. 2014). However, with the spread of orthogeriatric care models the clinical problem of postoperative delirium is being paid increasing attention. Elderly patients with hip fractures are especially vulnerable to POD, leading to poor functional recovery (Marcantonio et al. 2000). While patients with fragility fractures can already profit from interdisciplinary orthogeriatric care models, the topic still seems underrepresented in elective orthopedic surgery. Studies dealing with the relationship of POD to other complications after elective THA and TKA are rare (Aziz et al. 2018) and, to the best of our knowledge, no study has previously evaluated the impact of POD on patient-reported outcome measures (PROMs) after THA or TKA.

Therefore, we retrospectively evaluated the impact of postoperative delirium on complications and patient-reported outcome in a consecutive series of 10,140 patients who had undergone primary elective THA or TKA at a high-volume arthroplasty center. We hypothesized that patients who exhibit POD have higher rates of complications and worse PROM than patients without POD.

## Patients and methods

### Study design and study population

This is a retrospective study based on a database derived from the hospital information system and the department’s joint registry. From the database, all patients who had undergone primary elective THA and TKA between 2011 and 2020 were included, representing a consecutive series. As capture of patient-reported outcome measures (PROM) did not start until the establishment of a certified arthroplasty center in October 2012 and some patients were lost to follow-up 1 year postoperatively, PROMs were only available for a subgroup of patients.

Reoperation within 90 days, readmission within 90 days, complications, and transfusion were analyzed. Complications were categorized into surgical (major bleeding with need for transfusion, periprosthetic fracture, wound healing disorder, wound infection, dislocation) and non-surgical (myocardial infarction, decompensated heart failure, cardiac arrhythmias, pneumonia, renal failure, urinary tract infection, collapse, thrombosis, pulmonary embolism, cerebrovascular accident). Furthermore, complications were categorized according to the Clavien–Dindo classification (Dindo et al. 2004). This classification system ranks complications in 5 grades, based on the therapy used for correction. Any deviation from the normal postoperative course without the need for pharmacological treatment or surgical, endoscopic, and radiological intervention represents a Grade I complication. Grade II complications require specific pharmacological treatment, whereas Grade III complications result in surgical, endoscopic, or radiological intervention. Grade IV complications are defined as life-threatening events requiring intensive care management. Grade V represents the death of a patient (Dindo et al. 2004). Follow up for the parameter “surgical complication” was limited to 30 days postoperatively. Non-surgical and Clavien–Dindo IV° complications could only be captured during hospital stay (mean 9 days postoperatively).

### Surgical techniques

Indications for surgery were primary or secondary end-stage hip or knee osteoarthritis. All operations were performed in a single Department of Orthopedic Surgery at a University Medical Center. All patients received the same standardized treatment protocol for THA or TKA respectively. THA was performed under spinal anesthesia, whereas TKA was performed using anesthesia with perineural catheters. Only if there are contraindications for regional anesthesia or in the case of explicit patient desire was general anesthesia applied. Cementless THA was performed in the lateral decubitus position. A minimally invasive anterolateral approach was used. Cemented TKA was performed through a medial parapatellar approach. No patellar resurfacing was performed. Postoperative pain management included non-steroidal anti-inflammatory drugs and retarded opioids if needed. Mobilization was started on the first day following surgery under full or partial weight-bearing, according to the instructions of the orthopedic surgeon.

### Data collection (Figure 1, see Supplementary data)

Diagnoses coded at the time of hospitalization and discharge were extracted from the hospital information system (ORBIS; Agfa Healthcare, Mortsel, Belgium) including corresponding ICD-10 codes. Diagnostic codes had been entered by professional clinical coders and were double-checked by physicians using information gathered from patients’ medical records. Postoperative delirium was coded when the Nursing Delirium Screening Scale (Nu-DESC) was greater or equal to 2 as proposed by Gaudreau et al. (2005). The Nu-DESCs were assessed daily by skilled nursing staff. Complications were assessed according to the ICD-10 codes at the time of discharge. Further available data from our clinical information system were age, sex, operative procedure, length of stay, transfusion, transfer to intensive care unit, reoperation, and readmission. Patient-reported outcome were extracted from the department’s joint registry. The Western Ontario and McMaster Universities Arthritis index (WOMAC) was assessed preoperatively and 1 year postoperatively. Responders were differentiated from non-responders by means of the criteria of the Outcome Measures in Rheumatology and Osteoarthritis Research Society International consensus (OMERACT-OARSI) (Pham et al. 2004). The OMERACT-OARSI criteria identify patients as responders after THA or TKA if the WOMAC index shows an improvement in pain or function, either relatively by at least 50% or absolutely by at least 20 points. Alternatively, patients are defined as responders if 2 of the following criteria are met: Improvement of the pain subscore by at least 20% and at least 10 points, improvement of the function subscore by at least 20% and at least 10 points, or improvement in the global index by at least 20% and at least 10 points (Pham et al. 2004).

### Statistics

Group comparisons were performed by 2-sided t-tests. Absolute and relative frequencies were given for categorical data and compared between groups by chi-square tests. The hypotheses of the study were tested on 5% significance level. Multivariable logistic regression analyses were conducted to provide risk factor estimates adjusted for confounding bias in terms of reoperation, readmission, and Clavien–Dindo IV° complications while controlling for other variables known to be associated with adverse surgical outcomes. According to the literature, surgery site knee, long operative time, male sex, increasing age, and high ASA classification are known risk factors for complications after THA and TKA (Weber et al. 2018). Adjustment of covariates was performed based on considerations regarding cause–effect. The 6-step approach was used to prevent adjustment bias (Shrier and Platt 2008). The assumed cause–effect relation is shown in Figure 2 (see Supplementary data). Confidence level was defined at 95% and is presented as confidence interval (CI). IBM SPSS Statistics 25 (IBM Corp, Armonk, NY, USA) was used for analysis.

### Ethics, funding, and potential conflicts of interest

This study was approved by the Ethics Committee of the University Hospital Regensburg, Germany (20-1821-104). Informed consent was not necessary for this type of study. No funding was received. The authors have no conflicts of interest to declare.

## Results

**Figure 3.**
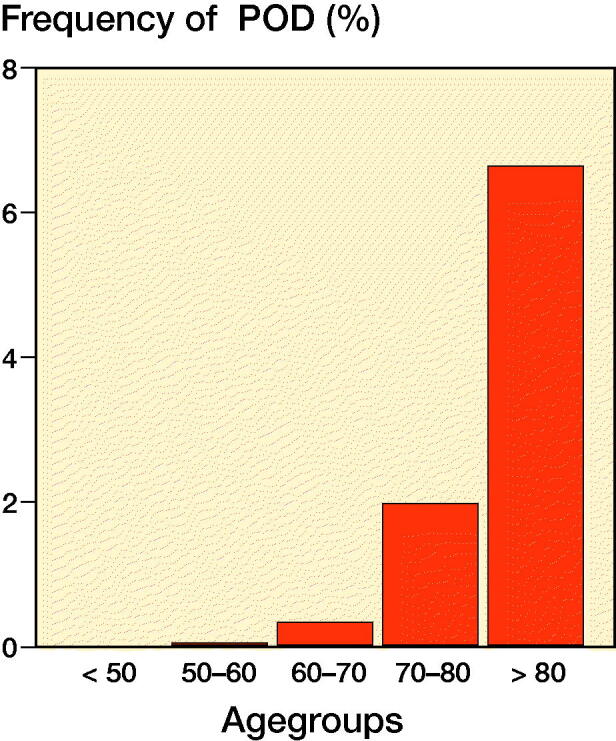
Age distribution of postoperative delirium (POD) in patients undergoing primary elective THA or TKA.

There were 5,575 and 4,565 patients who underwent THA or TKA, respectively, during the study period. Patient-reported outcome measures up to 1 year postoperatively were available for a subgroup of 4,189 patients (Table 1). Incidences of POD in the study group and the PROM subgroup were 1.4% and 1.2%, respectively. Mean age in the POD cohort was 79 years (SD 6.8) versus 66 years (SD 11) in the non-POD cohort. The distribution of POD in the study group according to patient age is shown in Figure 3.

**Table 1.** Characteristics of study group and subgroup with available patient-reported outcome measures (PROMs). Values are percentage unless otherwise specified

	Study group	Non-POD cohort	POD cohort	PROM subgroup
Demographics	n = 10,140	n = 10,001	n = 139	n = 4,189
Age **^a^**	66 (11)	66 (11)	79 (7)	66 (10)
Female sex	58	58	53	56
Total hip arthroplasty	55	57	58	55
Operative time, minutes **^a^**	77 (30)	76 (30)	81 (32)	75 (34)
Length of stay, days **^a^**	9 (4)	9 (4)	10 (6)	9 (2)
ASA classification 1	13	13	2	15
ASA classification 2	57	57	43	58
ASA classification 3	31	30	54	27
ASA classification 4	0.3	0.3	0.7	0.2
CCI **^a^**	0.5 (0.9)	0.4 (0.9)	2 (1)	0.4 (0.8)
Dementia	0.1	0.1	1	0
Cerebrovascular disease	2	2	7	2

a Values are mean (standard deviation).

ASA = American Society of Anesthesiologists.

CCI = Charlson Comorbidity Index.

**Figure 4.**
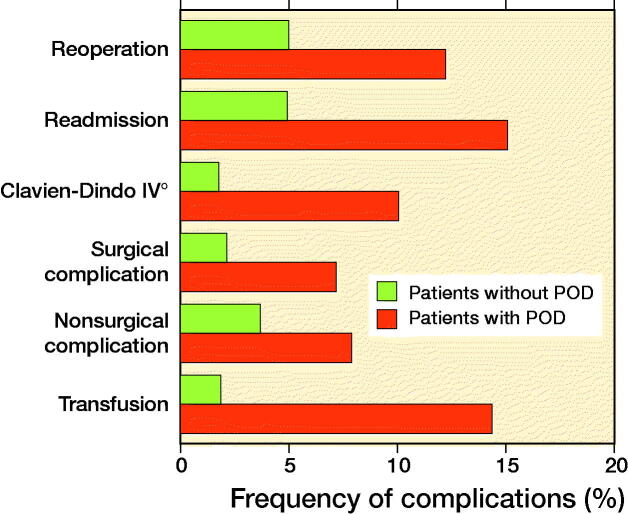
Rates of complications in patients with and without postoperative delirium (POD) after primary elective total hip or knee arthroplasty.

Patients who exhibited POD showed increased rates of reoperation (12% vs. 5%, p < 0.001) and readmission (15% vs. 5%, p < 0.001) compared with patients without POD (Table 2; Figure 4).

**Table 2.** Complications after total hip or knee arthroplasty in patients with and without postoperative delirium. Values are count (%)

Adverse event	Non-POD cohort n = 10,001	POD cohort n = 139	p-value
Reoperation within 90 days	503 (5.0)	17 (12)	< 0.001
Readmission within 90 days	495 (4.9)	21 (15)	< 0.001
Surgical complications	215 (2.1)	10 (7)	< 0.001
Non-surgical complications	370 (3.7)	11 (8)	0.009
Clavien–Dindo IV°	177 (1.8)	14 (10)	< 0.001
Transfusion	185 (1.8)	20 (14)	< 0.001
PROM subgroup	4,139	50	
Responder rate	3,602 (87)	38 (76)	0.02

POD = postoperative delirium.

The rates for surgical, non-surgical, and Clavien–Dindo grade IV complications were higher in the POD cohort, compared with the non-POD cohort (p < 0.01). Transfusion rate was 13% higher in patients with POD compared with patients without POD (p < 0.001; Table 2).

Patients with POD showed a decreased responder rate compared with patients without POD (76% vs. 87%; p = 0.02) 1 year postoperatively (Table 2).

The results largely held true for subgroup analyses of THA and TKA (Tables 3 and 4, see Supplementary data).

**Table 5.** Multivariable analysis with odds ratio (OR) and 95% confidence interval (CI) for total effect of postoperative delirium on reoperation, readmission, and Clavien–Dindo IV° complications after total joint arthroplasty

Variable	OR (CI)	p-value
Reoperation		
Postoperative delirium	2.0 (1.1–3.4)	0.02
Surgery site (knee)	0.9 (0.8–1.1)	0.3
Age per 5 years	1.1 (1.0–1.1)	0.04
ASA classification	1.6 (1.4–1.9)	0.001
Operative time per 15 minutes	1.3 (1.2–1.3)	0.001
Sex (male)	0.9 (0.7–1.1)	0.1
Readmission		
Postoperative delirium	2.4 (1.5–4.0)	0.001
Surgery site (knee)	1.1 (0.9–1.4)	0.2
Age per 5 years	1.1 (1.0–1.2)	0.001
ASA classification	1.6 (1.4–1.9)	0.001
Operative time per 15 minutes	1.2 (1.2–1.3)	0.001
Sex (male)	0.9 (0.8–1.1)	0.6
Clavien–Dindo IV		
Postoperative delirium	2.7 (1.5–5.0)	0.001
Surgery site (knee)	0.9 (0.6–1.2)	0.4
Age per 5 years	1.4 (1.3–1.5)	0.001
ASA classification	1.8 (1.4–2.3)	0.001
Operative time per 15 minutes	1.1 (1.0–1.2)	0.01
Sex (male)	0.8 (0.6–1.2)	0.3

ASA = American Society of Anesthesiologists.

For assumed cause–effect relation please see Figure 2.

### Multivariable logistic regression analysis

Multivariable logistic regression analysis identified POD as independent risk factors for reoperation (OR = 2, CI 1–3), readmission (OR = 2, CI 2–4), and Clavien–Dindo IV° complications (OR = 3, CI 2–5; Table 5).

## Discussion

We found that patients with POD showed higher rates of all captured complications after THA and TKA. Furthermore, patients who exhibited POD were more likely to be non-responders compared with patients without POD up to one year postoperatively. Multivariable logistic regression analysis identified POD as risk factor for reoperation, readmission, and Clavien–Dindo IV° complications. In our study the incidence of POD was 1.4%. This is consistent with the existing literature as recent retrospective analyses found incidences of POD from 0.68% to 2.21% (Aziz et al. 2018, Weinstein et al. 2018, Yang et al. 2020). A prospective study of 6,331 elective THA and TKA patients aged 70 years or older found an incidence of 0.7% for POD (Petersen et al. 2017). In 2015, a meta-analysis of prospective studies described incidences of POD from 0% to 10% after total joint arthroplasty and concluded that reliable estimation of the incidence of POD is challenging, as study groups are heterogenic and a variety of assessment tools are used (Bin Abd Razak and Yung 2015). Even higher incidences up to 42% are reported for patients undergoing THA because of hip fractures (Mosk et al. 2017). As pre-existing dementia, comorbidities, and age are well-known risk factors, it is not surprising that patients with fragility fractures are especially prone to POD. In our study, mild and especially hypoactive forms of POD might not have been recognized appropriately (Inouye et al. 2014). Consequently, the true incidence of POD after elective THA and TKA might be higher than assumed and could only be determined in a prospective controlled setting.

Whereas incidences and risk factors of POD are well reported, less is known about the associations between POD and other postoperative complications after THA and TKA. In our study, reoperation and readmission rate within 90 days after surgery for patients with POD was 2 to 3 times higher than for patients without POD. The proportion of patients with surgical complications, non-surgical complications, Clavien–Dindo IV° complications, and transfusion was even 2 to 7 times higher in the cohort with POD. A recent retrospective analysis also found a 2- to 3-fold increase of surgical complications and a 2- to 4-fold increase of non-surgical complications after primary elective THA (Aziz et al. 2018). Multivariable logistic regression analyses showed independent associations of POD with 90-day reoperation, 90-day readmission, and Clavien–Dindo IV° complications, while controlling for age, ASA classification, and operative time. Our results are confirmed by Aziz et al. (2018), who found that POD was independently associated with major (OR 2.0; CI 1.7–2.4; p < 0.001) and minor (OR 2.0; CI 1.7–2.4; p < 0.001) complications, while controlling for age, sex, and number of comorbidities. The data show that exhibition of POD favors postoperative complications. However, a reverse effect might also be present as major complications, especially those requiring ICU management, put patients at higher risk of POD (Aldecoa et al. 2017). Overall, assessment of causal relationships between POD and other complications is complex (Figure 2, see Supplementary data). Unfortunately, our database provides no information concerning the temporal relation between documentation of POD and occurrence of complications. Demographic analysis of our study cohorts showed higher comorbidity burden and incidences of dementia or cerebrovascular disease in the POD cohort (Table 1). Hence, the occurrence of POD and postoperative complications could also be interpreted as a consequence of underlying comorbidity (Meyer et al. 2020). We tried to investigate the independent effect of POD on complications after THA and TKA by use of multivariable regression analysis. Controlling for comorbidity measured by ASA score, POD was independently associated with complications after THA and TKA. Nevertheless, due to the complex relations of POD, comorbidity, and postoperative complications, the results of this study have to be interpreted with caution. To the best of our knowledge, this is the first study to evaluate PROMs in patients with POD after THA and TKA. The overall responder rate as defined by the OMERACT-OARSI criteria was 91% and 82%, respectively. This is consistent with recent literature, where responder rates of 86% to 93% for THA and 73% to 86% for TKA are described (Judge et al. 2010, Weber et al. 2018, Overgaard et al. 2019). We found a significantly worse responder rate in patients with POD compared with patients without POD. Comparative data are hardly available. Abelha et al. (2013) reported lower quality of life for patients who experienced POD during ICU treatment after major surgery at follow-up 6 months postoperatively. With regard to orthopedic surgery, Duppils and Wikblad (2000) found that patients with POD after hip fracture scored lower in PROMs at follow-up than those without delirium. Demographic analysis of our study cohorts showed higher age and increased comorbidity burden in the POD cohort (see Table 1). Hence, a possible influence of age and comorbidity on responder rate has to be taken in account. In a former study, comorbidity was independently associated with responder rate, whereas age was not (Weber et al. 2018). Although PROMs were available only for a subgroup of patients, we assume that the results of the subgroup are valid, as demographic characteristics of the study group and the subgroup with available PROMS were comparable (see Table 1). Furthermore, incidences of POD and overall responder rates in the subgroup were consistent with current literature (Aziz et al. 2018, Weber et al. 2018).

Our findings underline the relevance of POD in primary elective THA and TKA. Patients who experience POD are prone to other major complications and have worse patient-reported outcome up to 1 year postoperatively. Therefore, POD prophylaxis and thorough screening of at-risk patients is mandatory to reliably recognize POD and enable early initiation of adequate treatment.

Our study has some limitations. Data acquisition was limited to the data available from the hospital information system and the institutional joint registry. Because of the limited number of POD patients in the study group our results need to be confirmed in larger cohorts. Mild and especially hypoactive forms of delirium might have not been recognized appropriately and the severity of POD was not captured. Due to the retrospective design, analysis of temporal relation of POD and occurrence of complications was not possible. Follow-up for the variable surgical complication was limited to 30 days postoperatively. Non-surgical and Clavien–Dindo IV° complications could only be captured during hospital stay (mean 9 days postoperatively). Some patients might have also been readmitted to other hospitals. Furthermore, other parameters with possible impact on outcome, such as BMI and psychosocial aspects, could not be captured. Another limitation is the mean length of stay in our cohort. Considering international standards, the comparatively long length of stay is related to the German healthcare system and represents the typical length of stay after THA and TKA during the study period. Despite these limitations, our results emphasize the relationship of POD to complications after THA and TKA. Furthermore, our study is the first to demonstrate a negative impact of POD on patient-reported outcome after elective total joint arthroplasty. A further strength of our study is the high number of patients in a monocentric study design that guarantees standardized operative workflows and postoperative treatment protocols for THA and TKA. In this way, possible confounding factors were minimized. Future research should focus on the utility of orthogeriatric care models in prevention of POD and its negative consequences.

## Conclusion

The risk of postoperative delirium should be taken seriously in elderly patients undergoing primary elective THA and TKA. Even though cause and effect are difficult to differentiate, affected patients obviously suffer more complications and show worse PROMs up to 1 year postoperatively. Prevention strategies, screening for at-risk patients, and standardized therapy protocols are mandatory to avoid burden on patients and healthcare providers.

## Supplementary Material

Supplemental Material

## Supplementary data

Figures 1–2 and Tables 3–4 are available as supplementary data in the online version of this article, http://dx.doi.org/10.1080/17453674.2021.1980676

GM, MW, TK, and MM originated the idea for the study and led on its design. MW, TK, and JG supervised the project. MW, MM, JUG, LP, and TK participated in the design of the study and were responsible for data acquisition. GM, TR, MW, and MM contributed to analysis and interpretation of data. MW provided statistical consultation. MM drafted the manuscript. TK, GM, JG, and MW revised the manuscript critically for important intellectual content. All authors read and approved the final version of the manuscript.

*Acta* thanks Esa Jämsen and Annette W-Dahl for help with peer review of this study.
