# Supplementary material for: Postoperative delirium is a risk factor for complications and poor outcome after total hip and knee arthroplasty
Source: Acta Orthop. 2021 Oct 5;92(6):695–700. doi: 10.1080/17453674.2021.1980676 (PMC8635535; doi:10.1080/17453674.2021.1980676)
Supplement: Supplemental Material [file IORT_A_1980676_SM6097.pdf]

Supplementary data

Table 3. Adverse events after total hip arthroplasty in patients with and without postoperative delirium. Values are count (%)

Adverse event	Non-POD cohort n = 5,495	POD cohort n = 80	p-value
Reoperation within 90 days	274 (5.0)	10	0.002
Readmission within 90 days	247 (4.5)	10	0.001
Surgical complications	138 (2.5)	6	0.005
Non-surgical complications	188 (3.4)	7	0.01
Clavien–Dindo IV°	96 (1.7)	10	< 0.001
Transfusion	106 (1.9)	13	< 0.001
PROM subgroup	2,289	25	
Responder rate	2,088 (91)	20	0.05

POD = postoperative delirium.

Table 4. Adverse events after total knee arthroplasty in patients with and without postoperative delirium. Values are count (%)

Adverse event	Non-POD cohort n = 4,506	POD cohort n = 59	p-value
Reoperation within 90 days	229 (5.1)	7	0.02
Readmission within 90 days	248 (5.5)	11	< 0.001
Surgical complications	77 (1.7)	4	0.003
Non-surgical complications	182 (4.0)	4	0.2
Clavien–Dindo IV°	81 (1.8)	4	0.005
Transfusion	79 (1.8)	7	< 0.001
PROM subgroup	1,850	25	
Responder rate	1 514 (82)	18	0.2

POD = postoperative delirium.

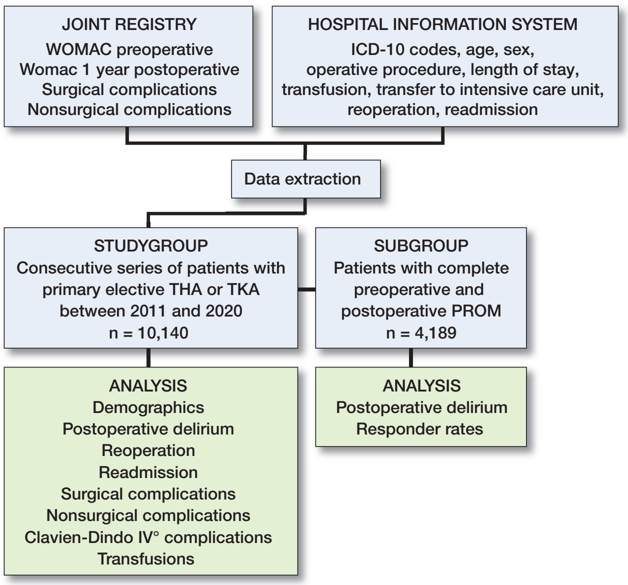

Figure 1. Data collection and analysis flow chart.

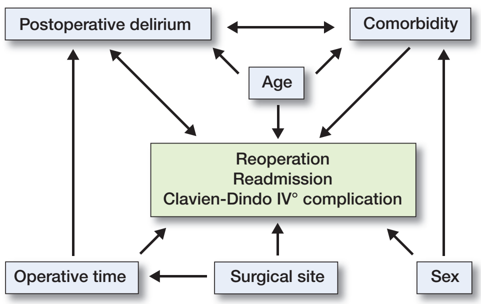

Figure 2. Assumed cause–effect interactions.
